# A low light video enhancement using interval valued intuitionistic fuzzy set with HVI space

**DOI:** 10.1038/s41598-025-34274-y

**Published:** 2026-01-28

**Authors:** M. Manivasagan, S. Jagatheswari

**Affiliations:** Department of Mathematics, School of Advanced Sciences, Vellore Institute of Technology, Vellore, Tamilnadu 632014 India

**Keywords:** Interval valued intuitionistic fuzzy set, Entropy, Video enhancement, No-reference metrics, HVI space, Engineering, Health care, Mathematics and computing

## Abstract

Enhancing low-light videos is essential for applications such as surveillance, autonomous driving, and medical imaging. However, achieving effective contrast improvement remains challenging due to poor illumination, noise, and over-enhancement artifacts. To address these issues, this study presents a novel Interval-Valued Intuitionistic Fuzzy Generator (IVIFG)-based framework for low-light video enhancement. In the proposed approach, the input video is decomposed into individual frames, which are enhanced using the IVIFG model. The enhanced frames are then transformed into the HVI color space, and visually optimal frames are selected using an entropy-based criterion to preserve illumination, contrast, and perceptual color fidelity. To demonstrate practical applicability, the method is applied to a no-reference low-light video and evaluated using standard quality metrics, including entropy, AMBE, CII, NIQE, and BRISQUE. Additional validation is performed on a custom low-light traffic dataset. In both scenarios, the proposed approach is compared with conventional fuzzy methods (IFA, IVIFA+CLAHE, ANV, NIFG) and deep-learning models (Zero-DCE, Zero-DCE++, EFINet, and FlightNet). The results show that the IVIFG framework consistently outperforms existing techniques, achieving superior performance across all no-reference metrics. These findings highlight its robustness and strong potential for real-world deployment in low-light video enhancement applications.

## Introduction

Video enhancement is a fundamental task in image and video processing, aiming to restore visibility and improve visual clarity for reliable interpretation. It plays an essential role in applications such as surveillance, remote sensing, medical diagnostics, multimedia analysis, and autonomous navigation. Low-light video enhancement (LLVE) remains particularly challenging due to illumination inconsistency, noise amplification, color distortion, and the need to preserve temporal stability across frames. These challenges become more severe in dynamic scenes, where visibility degradation directly impacts downstream tasks such as detection, tracking, measurement, and summarization.

Conventional enhancement strategies have addressed LLVE from multiple perspectives. Rahman et al.^1^ proposed a visibility enhancement framework combining white balance correction, probabilistic illumination modeling, and multiscale fusion. Retinex models^2,3^ improved illumination–reflectance decomposition, but its performance degraded under strong illumination variation. Transformer-based methods such as STA-SUNet^4^ and ALAT^5^ effectively captured spatio-temporal dependencies and local structural alignment, yet required large paired datasets and high computational resources, limiting their deployment under noisy or real-time conditions but often failed to preserve fine textures in severely dark regions.

Unsupervised LLVE techniques attempt to eliminate reliance on paired datasets. Ye et al.^6^ developed a spatio-temporal propagation network to reduce flicker. while Wen et al.^7^ introduced a multi-scale Retinex-based unsupervised model with improved stability. However, both approaches remain sensitive to illumination fluctuations and require complex training pipelines. Color-space-based studies, including Lab* segmentation by Wang et al.^8^ and Retinex–VGG fusion by Lee et al.^9^, demonstrated perceptual gains, yet nonlinear distortions and color-coupling issues persisted in extreme low-light conditions. Broader video-processing domains further highlight the need for robust LLVE. In low-light photography, LumiNet^10^ improved visibility through multispatial attention and GAN refinement, while Bose et al. introduced LoLTV^11^ for traffic surveillance and demonstrated the importance of reliable illumination correction for high-level analytics. Mehra et al.^12^ showed that crowd monitoring accuracy heavily depends on stable brightness estimation. Video summarization models such as GenSumNet^13^ also emphasized the need for illumination-stable inputs to maintain summarization accuracy.

Event-driven methods^14,15^ improved temporal fidelity but relied on specialized event cameras, restricting real-world usage. Recent wavelet and diffusion-based models, including Lin et al.^16^ and Peng et al.^17^, enhanced temporal consistency and training flexibility yet struggled under high noise or complex motion. Application-specific LLVE solutions−respiratory rate estimation under low light^18^, enhancement in sand-dust environments^19^, and real-time smartphone-based enhancement via VLight^20^ demonstrated strong performance but remained limited by illumination instability, particulate interference, or simplified model assumptions.

Fuzzy logic has been explored as a mathematically grounded alternative for modelling uncertainty in dark regions. IFS-based fusion methods improved luminance preservation for still images^21^, while temporal intuitionistic fuzzy sets^22^ enhanced videos but lacked interval-valued modelling. Chinnappan et al.^23^ applied intuitionistic fuzzy generators (IFG) with entropy-based enhancement and CLAHE for video frames; however, their approach processed each frame independently, did not explicitly reduce uncertainty, and relied on CLAHE, which increases computational complexity and may affect natural appearance. Reviews by Ye et al.^24^ highlighted that existing LLVE methods still suffer from instability and limited adaptability under severe low-light conditions. More recent interval-valued fuzzy approaches^25–28^ improved contrast and detail recovery for images but were not extended effectively to video, and most relied on unstable color models such as HSV. Yan et al.^29^ introduced the HVI color space to improve low-light perception but required deep learning architectures and paired datasets.

Collectively, these studies reveal persistent challenges:

1. Ambiguous intensity regions remain difficult to model with conventional or deep models;
2. Temporal consistency is not reliably maintained across frames;
3. Existing fuzzy-logic methods lack interval-valued modelling and frame-adaptive tuning; and
4. Widely used color spaces (RGB, HSV, Lab*) remain unstable under extreme low-light conditions.

To address these limitations, this work proposes an interval-valued intuitionistic fuzzy generator (IVIFG) integrated with the HVI color space, providing a flexible and perceptually stable representation for low-light videos. The HVI model offers superior chromatic stability under low illumination, making it highly suitable for interval-valued fuzzy modelling. The proposed method extracts frames using the actual frame count obtained from MATLAB’s video reader, ensuring consistent, systematic, and data-driven frame selection. Furthermore, an entropy-driven $\lambda$– q adaptive tuning strategy generates multiple IVIF-enhanced frames and automatically selects the most informative one, achieving balanced illumination correction, improved color consistency, and stable temporal behavior.

The framework of the article is outlined in Sect. “Preliminaries”, which covers fundamental concepts related to fuzzy sets, their extensions, enhancement techniques, and color spaces. The proposed methodology is detailed in Sect. “Proposed methodology”. Section “Experimental analysis” provides the experimental evaluation, detailing the dataset description, the performance assessment of the proposed and comparative methods, and a comprehensive discussion of the results. The section also reports quantitative outcomes presented in tabular form, an analysis of the employed performance metrics, and an ablation study to examine the contribution of individual components of the method. Lastly, Sect. “Conclusion and future work” provides a summary of the research findings.

## Preliminaries

### Fuzzy set

A fuzzy set R in X is defined as^30^ a collection of ordered pairs as follows: Let X be the universe of discourse x $\in$ X,

1 $$\begin{aligned} R = \{(x, \mu _R(x)) : x \in X\} \end{aligned}$$

where $\mu _R: X \rightarrow [0,1]$ is called the membership function of x in R.

### Intuitionistic fuzzy set

$R^{*}$ in X is an intuitionistic fuzzy set defined as^31^

2 $$\begin{aligned} R^{*} = \{\langle x, \mu _{R}(x),\nu _{R}(x) \rangle | x \in X\} \end{aligned}$$

where $\mu _{R}(x)\rightarrow [0,1],\nu _{R}(x)\rightarrow [0,1]$ are the belongingness and non-belongingness degrees of an element x in $R^{*}$ with the condition $0 \leqslant \mu _{R}(x) + \nu _{R}(x) \leqslant 1$.

### Interval-valued intuitionistic fuzzy sets (IVIFSs)

An IVIFS $\phi (R)$ over X can be expressed as^32^

3 $$\begin{aligned} \phi (R) = \{\langle x, X_{\phi (R)}(x), Y_{\phi (R)}(x) \rangle | x \in X\} \end{aligned}$$

where $X_{\phi (R)}(x)$ $\text { and }$ $Y_{\phi (R)}(x)\subset [0,1]$ are intervals for both members and non-members, respectively, and $\sup X_{\phi (R)}(x) + \sup Y_{\phi (R)}(x) \le 1, \text { for all } x \in X.$

Consider the mapping $\phi : IFS \rightarrow IVIFS$ described as

$$\phi (R) = \{ x, [X_{\phi (R)}^L(x), X_{\phi (R)}^U(x)],[Y_{\phi (R)}^L(x),$$

$$Y_{\phi (R)}^U(x)] \}$$, $$\forall x \in X$$

where $$X_{\phi (R)}^L(x) = \mu _R(x) - k_1 \cdot \pi _R(x), \quad 0 \le k_1 \le \frac{\mu _R(x)}{\pi _R(x)}$$

$$X_{\phi (R)}^U(x) = \mu _R(x) + k_2 \cdot \pi _R(x), \quad 0 \le k_2 \le 1$$

$$Y_{\phi (R)}^L(x) = \nu _R(x) - k_3 \cdot \pi _R(x), \quad 0 \le k_3 \le \frac{\nu _R(x)}{\pi _R(x)},$$

$$Y_{\phi (R)}^U(x) = \nu _R(x) + k_4 \cdot \pi _R(x), \quad 0 \le k_4 \le 1$$

with the conditions:

$$0 \le k_2 + k_4 \le 1, \quad 0< k_1 + k_2 \le 1, \quad 0 < k_3 + k_4 \le 1$$

The following is how we define the membership and non-membership widths:

4 $$\begin{aligned} H_M&= X_{\phi (R)}^U(x) - X_{\phi (R)}^L(x) = (k_1+k_2) \cdot \pi _R(x) \end{aligned}$$

5 $$\begin{aligned} H_N&= Y_{\phi (R)}^U(x) - Y_{\phi (R)}^L(x) = (k_3 + k_4) \cdot \pi _R(x) \end{aligned}$$

### RGB space

The RGB color space^33^ is one of the most fundamental and widely used models in image processing. It represents images by combining different intensities of red, green, and blue light to produce a broad spectrum of colors. However, for low light image enhancement, the RGB model has limitations due to the weak correlation between its three channels. Even small variations in one channel may cause noticeable shifts in brightness and color balance, making RGB less suitable for processing images captured under poor illumination.

### HSV space

The HSV color space^25^ is frequently employed in image processing owing to its intuitive structure and computational efficiency. Unlike RGB, where brightness and chromaticity are entangled, HSV decouples these components, thereby enabling more precise adjustments. In many enhancement tasks, only the value (V) component requires processing, reducing computational cost compared with RGB, which involves all three channels. Furthermore, HSV reduces inter-channel correlation and provides greater robustness to illumination changes, making it well suited for applications such as enhancement, segmentation, and feature extraction.

### HVI space

The method^29,34^ is inspired by established color spaces such as HSV (Hue–Saturation–Value), which is widely employed in image processing for its ability to separate chromatic components from intensity information. To enhance low light video frames while preserving color fidelity, a newly designed HVI (Hue–Vertical–Intensity) color space is adopted. Unlike HSV, which may introduce non-linear distortions and artifacts under extreme illumination due to its sensitivity to brightness variations and limited perceptual uniformity, the HVI model provides greater stability in low light scenarios. By representing color information in a geometrically interpretable manner and explicitly separating perceptual brightness from chromatic characteristics, HVI enables more accurate and consistent enhancement of both color appearance and structural details under challenging illumination conditions.

The HVI color space decomposes a normalized RGB image into three distinct components: a hue-related chromatic angle (H), a vertical chromatic magnitude (V), and a perceptual intensity (I). The conversion is defined as follows:

6 $$\begin{aligned} \theta =H=\frac{1}{\pi }arctan\left( \frac{\sqrt{3}(G-B)}{2R-G-B}\right) \end{aligned}$$

7 $$\begin{aligned} H=\frac{H+1}{2} \hspace{0.3cm} or \hspace{0.3cm} H=\frac{\theta +\pi }{2\pi } \end{aligned}$$

8 $$\begin{aligned} V=\sqrt{(R-G)^2+(R-B)^2\cdot (G-B)^2} \end{aligned}$$

9 $$\begin{aligned} I=0.2627(R)+0.6780(G)+0.0593(B) \end{aligned}$$

The intensity component is derived using coefficients aligned with the standard luminance conversion formula established in the ITU-R BT.2020 recommendation^35^. These coefficients account for the varying sensitivities of the human visual system to different wavelengths, with green contributing most significantly to perceived brightness, followed by red and then blue. This ensures that the intensity channel preserves perceptually relevant luminance information, which is critical for enhancing images in low light conditions.

The resulting HVI image is represented as

10 $$\begin{aligned} HVI(x,y) = [H(x,y), V(x,y), I(x,y)] \end{aligned}$$

where, H(x,y), V(x,y), I(x,y) denote the hue, vertical chromatic magnitude, and intensity at each pixel (x,y), respectively. This formulation enables localized contrast enhancement to be performed exclusively on the intensity channel while maintaining the hue and chromatic structure. In doing so, the HVI model effectively prevents color distortion and ensures natural visual appearance during low light enhancement.

### Shannon entropy

Shannon entropy^26^ is employed to quantify the informational content of an image. This method utilizes all the data present in the image to assess the level of uncertainty or probability associated with it. The calculation of entropy is represented by the following equation:

11 $$\begin{aligned} SE = -\sum _{i=1}^{x} \sum _{j=1}^{y} \bar{H}(i, j) \log \bar{H}(i, j) \end{aligned}$$

where *i* and *j* denote two different intensity values of the images, and $\bar{H}(i, j)$ represents the number of co-occurrences of *i* and *j*.

## Proposed methodology

In our method, the fuzzy membership, non-membership, and hesitation values are first computed using equations Eqs.(4) and (5). Unlike the conventional IFI model, where the enhanced intensity is defined as the sum of membership and hesitation, we regulate the hesitation

**Fig. 1:**
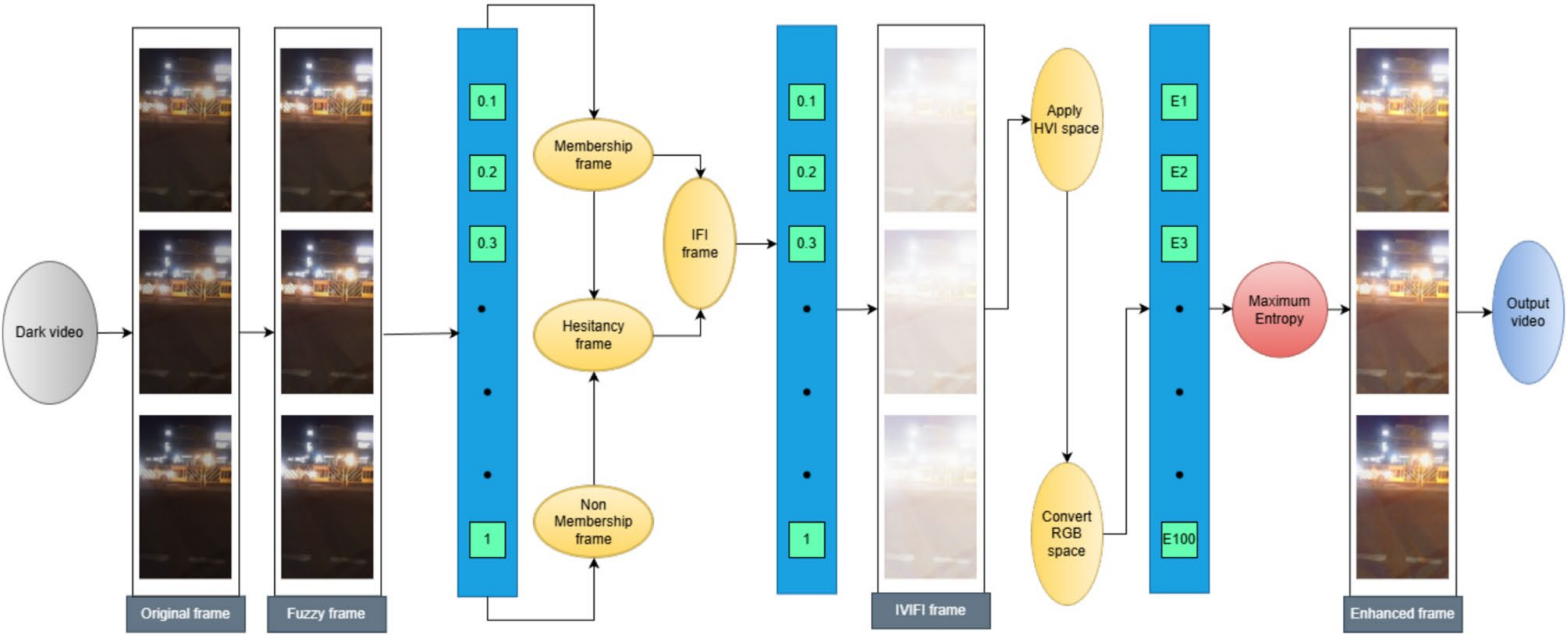
Flowchart of the proposed Interval-Valued Intuitionistic Fuzzy Generation (IVIFG–HVI) low-light video enhancement framework.

contribution by introducing a scaling parameter *q*. Specifically, for each $\lambda$ $\in$ [0.1, 0.2, …, 1.0] (10 values) and *q* $\in$ [0.1, 0.2, …, 1.0] (10 values), an interval-valued intuitionistic fuzzy image (IVIFI) is generated as

$$IVIFI_{\lambda ,q}$$ =$$\mu _\lambda$$+q.$$\pi _{\lambda }$$

This procedure produces 100 candidate IVIFI images corresponding to all $(\lambda ,q )$ combinations. Each candidate image is then mapped into the HVI color space and reconstructed back into RGB to obtain the enhanced output. For each output, the entropy value is computed, and the image with the maximum entropy is finally selected as the optimal enhanced result. This entropy-driven selection ensures that the proposed method retains the maximum amount of visual information while avoiding the over-enhancement effects that may arise from excessive hesitation contribution.

### Fuzzy image

Let I be an array of fuzzy singletons with dimensions M x N. An intuitionistic fuzzy set^36^ $R^{*}$ in S with the following definition of support

$$\sup (R^{*}) = \{ s \in S \mid \mu _R^{*}(s)> 0 \}$$

The picture ’I’ is fuzzified with the following mathematical equation^21^

12 $$\begin{aligned} \mu _{\bar{P}(\zeta (i, j))} = \frac{\bar{P}(\zeta _{ij}) - \bar{P}(\zeta _{min})}{\bar{P}(\zeta _{max}) - \bar{P}(\zeta _{min})} \end{aligned}$$

where $\bar{P}(\zeta _{ij})$ indicates the value of the (*i*, *j*)th pixel. $\bar{P}(\zeta _{max})$ and $\bar{P}(\zeta _{min})$ represent the highest and lowest pixel values of picture I, respectively.

### Novel interval valued intuitionistic fuzzy image

The low contrast video is converted into image frames. The frames are given as input and are changed into fuzzified images. Where the pixel values are normalized within [0, 1]. The proposed IVIFG algorithm is applied to the fuzzy image for enhancing low light video.

The interval-valued intuitionistic fuzzy set is an IFS extension. We construct the IVIFS as follows

13 $$\begin{aligned} \bar{N}(\mu _{\bar{P}}(\zeta )) = u^{-1}(u(1) - u(\mu _{P}(\zeta ))) \end{aligned}$$

Let us consider an increasing function as

14 $$\begin{aligned} u(\mu _{P}(\zeta )) =\frac{1}{(1+5e^{\lambda })}log(1+\mu _{P}(\zeta )(1+5e^{\lambda })), \quad \lambda> 0 \end{aligned}$$

where,

$$u(0) = \frac{1}{(1+5e^{\lambda })}log(1+0*(1+5e^{\lambda })) = 0$$ and

$$u(1) = \frac{1}{(1+5e^{\lambda })}log(1+1*(1+5e^{\lambda })) = \frac{1}{(1+5e^{\lambda })}log(2+5e^{\lambda }).$$

with the inverse function,

15 $$\begin{aligned} u^{-1}(\mu _{P}(\zeta )) =\frac{e^{\lambda *\mu _{P}(\zeta )}}{1+5e^{\lambda }} \end{aligned}$$

so,

$$\begin{aligned}&\bar{N}(\mu _{\bar{P}}(\zeta )) = u^{-1}\Bigg ( \frac{1}{(1+5e^{\lambda })}\log (2+5e^{\lambda }) \ - \frac{1}{(1+5e^{\lambda })} \log \big (1+\mu _{P}(\zeta )(1+5e^{\lambda })\big )\Bigg ) \end{aligned}$$

solving we get,

$$\bar{N}(\mu _{P}(\zeta )) = \chi (\mu _{P}(\zeta )) = \frac{1 - \mu _{P}(\zeta )}{1+\mu _{P}(\zeta )(1+5e^{\lambda })}$$, $$\quad \lambda> 0$$

where, $${\bar{N}}(1) = 0, {\bar{N}}(0) = 1$$.

By using IFS, the membership function of intuitionistic fuzzy set can be expressed as

16 $$\begin{aligned} \mu _{\bar{P}}(\zeta ) = 1 - \frac{(1 - \mu _{P}(\zeta ))}{(1 + \mu _{P}(\zeta )(1+5e^{\lambda }))} \end{aligned}$$

this implies that,

17 $$\begin{aligned} \mu _{\bar{P}}(\zeta ) = \frac{\mu _{P}(\zeta )(2+5e^{\lambda })}{1+\mu _{P}(\zeta )(1+5e^{\lambda })} \end{aligned}$$

The non-membership function of intuitionistic fuzzy set is expressed as

18 $$\begin{aligned} \nu _{\bar{P}}(\zeta ) = \frac{(1 - \mu _{P}(\zeta ))}{(1 + \mu _{P}(\zeta )(25e^{2\lambda }+20e^{\lambda }+3) )} \end{aligned}$$

and the indeterminacy function is expressed as

19 $$\begin{aligned} \pi _{\bar{P}}(\zeta ) = 1 - \mu _{\bar{P}}(\zeta ) - \nu _{\bar{P}}(\zeta ) \end{aligned}$$

where *t* $\in$ [0, 1]. The Interval valued function of intuitionistic fuzzy set is expressed as

20 $$\begin{aligned} IVIF=\mu _{\bar{P}}(\zeta )+H_M \end{aligned}$$

### Defuzzification

The process of converting a fuzzy number into crisp number is called defuzzification. The same process is followed for the defuzzification of the fuzzified images.

The defuzzification of IVIFI expression is as^37^ below

21 $$\begin{aligned} \zeta _{ij} = \mu _{ij} \cdot (\zeta _{max} - \zeta _{min}) + \zeta _{min} \end{aligned}$$

Therefore to defuzzify the fused image at the point (i, j). we use the following equation

22 $$\begin{aligned} \begin{aligned} {\textbf {Defuzzified Image}} = \text {IVIFI frame} \cdot (\zeta _{\max } - \zeta _{\min }) + \zeta _{\min } \end{aligned} \end{aligned}$$

**Algorithm 1:**
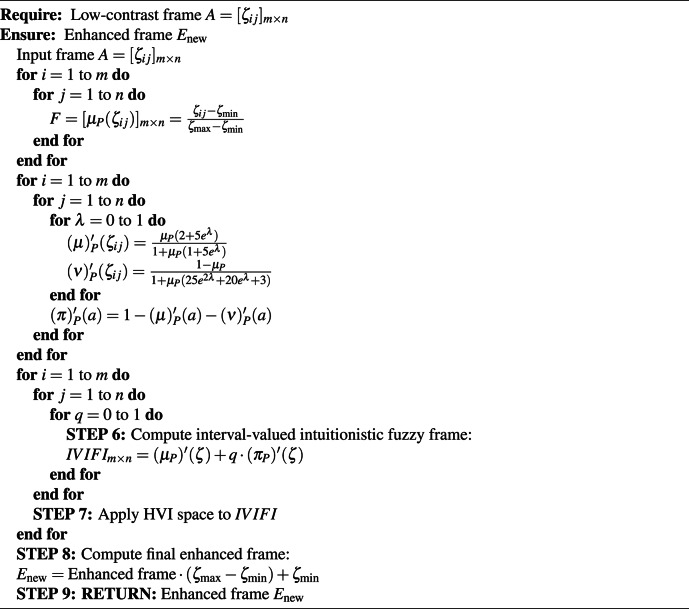
Pseudo-code of proposed method for enhancing dark video frames

## Experimental analysis

The experimental setup, datasets, and assessment measures are all thoroughly covered in this section, and comparative methodologies employed to validate the proposed enhancement framework. All experiments were executed on a Windows 11 machine equipped with 8 GB RAM. The proposed algorithm was implemented in MATLAB R2023a utilizing the Image Processing Toolbox.

For primary validation, we employed a low-light video recorded by the authors using a Moto G84 smartphone camera under natural dark lighting conditions. The original video sequence was recorded at standard frame rate and subsequently temporally trimmed to 4 seconds to meet the experimental requirements. The trimmed video was then decomposed into individual frames using MATLAB’s built-in video processing functions. From the extracted frames, 10 visually representative frames were selected for detailed qualitative and quantitative evaluation. Following enhancement, the processed frames were reassembled into a video sequence using MATLAB’s frame-to-video reconstruction functions to assess temporal consistency and visual coherence. This dataset provides a distinct illumination environment and dynamic scene motion, enabling a more diversified and comprehensive assessment of enhancement performance.

**Fig. 2:**
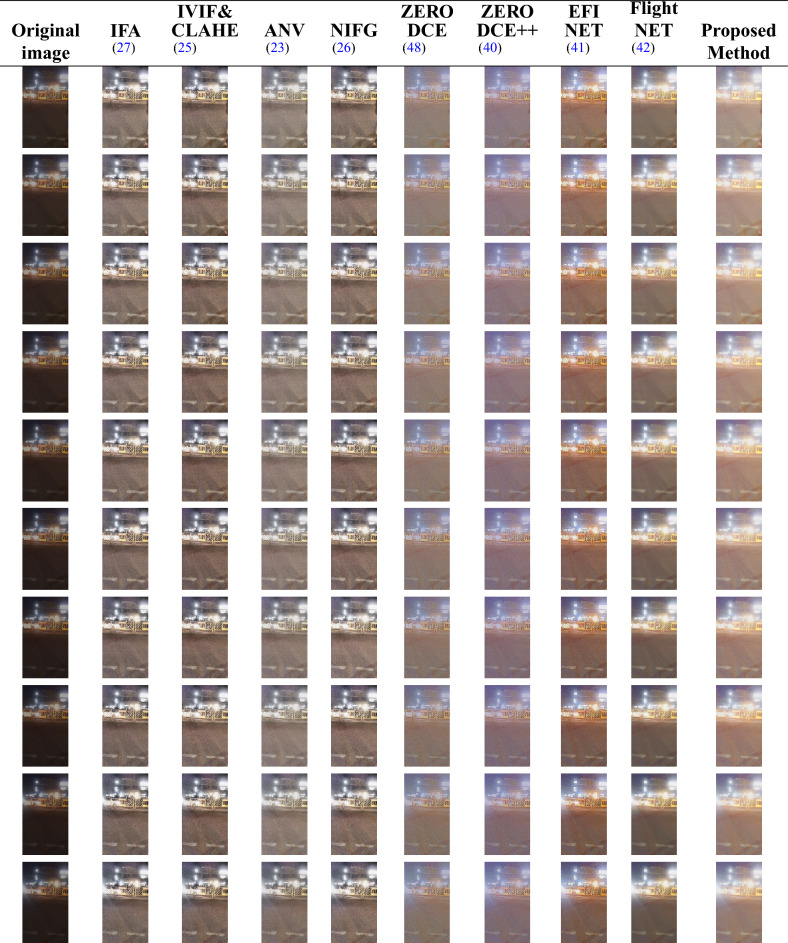
Sample frames extracted from a low-light video recorded by the authors using a Moto G84 smartphone camera. The frames illustrate the low-light characteristics used for primary evaluation.

###  Result and discussions

The overall workflow of the proposed methodology is depicted in Fig.1, which illustrates the sequential steps involved in enhancing low-light video frames. To evaluate the effectiveness of the proposed approach, it was benchmarked against a range of state-of-the-art deep learning and fuzzy-based enhancement methods, including Zero-DCE, Zero-DCE++, EFINet, and FlightNet. These methods were chosen because they represent leading approaches in both learning-based and fuzzy-based low-light image enhancement, providing a comprehensive comparison framework.

For qualitative assessment, a total of 121 frames were extracted from a 4-second low-light traffic video recorded by the authors using a Moto G84 smartphone, based on a frame rate of 30.03 frames per second. From these, 10 representative frames were selected and presented in Fig. 2 for side-by-side visual comparison. As seen in the figure, the proposed method delivers visually superior results, producing images with well-balanced illumination, enhanced structural clarity, and significantly reduced noise artifacts compared to the other methods. The improvements are particularly noticeable in challenging regions under low-light conditions, such as shadowed areas, reflective surfaces, and fine scene textures.

Quantitative evaluation was conducted using established image quality metrics including entropy, AMBE, CII, NIQE, and BRISQUE. These metrics assess different aspects of image quality, such as contrast enhancement, brightness preservation, naturalness, and perceptual quality. The results are summarized in tables (1, 2, 3, 4 and 5), where the best values are highlighted in italic and the second-best values in bold for ease of comparison. An ablation study was also performed to assess the contribution of each component in our framework, with metric values reported in table 6. In addition, Table 7 reports the average metric values computed over all 121 frames extracted from a 4-second segment of the low light video. Across all metrics, the proposed method either outperforms or remains competitive with state-of-the-art methods, demonstrating its robustness and effectiveness in enhancing low-light video frames while preserving important structural and textural details.

In addition to the enhancement quality, computational efficiency is a critical factor for practical applications, especially for real-time or near-real-time video processing. Table 8 provides a detailed comparison of the computational time required per frame by different methods. The proposed method demonstrates significantly reduced computational time compared to conventional fuzzy-based enhancement techniques, highlighting its suitability for practical deployment in real-world scenarios, such as traffic monitoring, surveillance, and autonomous driving, where processing speed is crucial.

Overall, the proposed approach combines high-quality visual enhancement with computational efficiency, making it a versatile and practical solution for diverse low-light video enhancement tasks. The consistent performance improvements across both qualitative and quantitative evaluations underline the method’s adaptability to different low-light environments and its potential advantage over existing enhancement strategies.

### Performance metrics

#### Absolute mean brightness error (AMBE)

The difference in average intensity between the original image and its enhanced version is assessed using AMBE (Average Modified Brightness Enhancement)^42^. This metric evaluates the effectiveness of an image enhancement method in maintaining brightness. A higher AMBE value typically signifies an image of superior quality, which suggests better preservation of brightness.

23 $$\begin{aligned} AMBE(O, E) = |mb(O) - mb(E)| \end{aligned}$$

where the mean brightness of the original and enhanced images are denoted by mb(O) and mb(E) correspondingly. Although some framess exhibit higher AMBE values than the recommended approach, the proposed method reliably surpasses earlier techniques, as shown in Table 2.

### Contrast improvement index (CII)

The Contrast Improvement Index (CII) is defined as^43^:

24 $$\begin{aligned} CII = \frac{C_{Enhanced}}{C_{Original}} \end{aligned}$$

in this context, $C_{Original}$ denotes the average intensity value of the original image, while $C_{Enhanced}$ indicates the average intensity value of the related enhanced image. A CII value exceeding 1 signifies an improvement in image contrast. The proposed method demonstrates superior performance compared to current solutions in most situations, as shown by the comparative results illustrated in Table 3.

#### Naturalness image quality evaluator(NIQE)

NIQE evaluates picture quality using a no-reference measure by measuring deviations from natural scene statistics (NSS) using a model built from pristine images. It operates without human-rated training, and lower scores indicate better quality. The metric is computed as^44^:

25 $$\begin{aligned} NIQE=\sqrt{(f_i-\mu _r)^T\left( \frac{\sum _r+\sum _i}{2}\right) ^{-1}(f_i-\mu _r)} \end{aligned}$$

**Table 1:** Entropy values corresponding to the enhanced frames shown in Fig.2.

Item no	Original image	IFA ^27^	IVIF& CLAHE ^25^	ANV ^23^	NIFG ^26^	ZERO DCE ^38^	ZERO DCE++ ^39^	EFI NET ^40^	Flight NET ^41^	Proposed method
1	6.4359	7.1324	7.1923	6.6636	7.0663	6.4674	6.4769	7.2447	6.9746	6.8269
2	6.4303	7.1030	7.1662	6.6193	7.0258	6.4713	6.5015	7.2320	6.9803	6.8885
3	6.4273	7.1046	7.1754	6.6139	7.0183	6.5246	6.5459	7.2526	7.0067	6.9557
4	6.3904	7.0913	7.1609	6.5996	7.0020	6.5142	6.5604	7.2259	6.9909	6.9747
5	6.3468	7.0718	7.1484	6.5783	6.9758	6.5510	6.5909	7.2234	6.9894	7.0253
6	6.2937	7.0606	7.1346	6.5846	6.9796	6.5310	6.5908	7.1863	6.9582	6.9262
7	6.2464	7.0554	7.1364	6.5831	6.9846	6.5585	6.6042	7.1791	6.9484	7.0024
8	6.2085	7.0444	7.1283	6.5661	6.9694	6.5820	6.6173	7.1700	6.9272	7.0452
9	6.2678	7.0422	7.1342	6.5584	6.9636	6.6353	6.6522	7.2329	6.9853	7.0316
10	6.2978	7.0237	7.1108	6.5441	6.9546	6.5985	6.6403	7.2354	7.0112	6.9847

**Table 2:** AMBE values corresponding to the enhanced frames shown in Fig.2.

Item no	IFA ^27^	IVIF& CLAHE ^25^	ANV ^23^	NIFG ^26^	ZERO DCE ^38^	ZERO DCE++ ^39^	EFI NET ^40^	Flight NET ^41^	Proposed method
1	69.0513	64.1018	**80.1967**	68.3756	76.8582	77.9681	77.9914	77.6875	*109.2807*
2	69.0366	63.9292	**80.2935**	68.1378	77.0043	77.8503	77.2114	79.4154	*108.6994*
3	68.9315	63.4840	**80.0405**	70.0562	77.4088	77.9006	76.0439	81.8536	*107.9615*
4	68.7616	63.0889	**80.1397**	69.8154	77.7167	77.9406	75.2874	80.4526	*107.1248*
5	68.6103	62.4946	**82.3987**	69.0508	78.1712	77.9683	73.9638	78.9607	*105.8684*
6	68.2672	62.0882	**82.3511**	71.0937	78.3603	77.9594	73.3956	74.8589	*104.6423*
7	68.1473	68.9199	82.1567	**84.7712**	78.5832	77.9878	72.3593	73.4003	*103.5851*
8	67.8802	75.2672	**87.1585**	77.0812	78.6317	77.9894	71.3225	72.5532	*102.7176*
9	67.3023	67.6712	**86.7710**	76.4827	78.4292	77.9602	71.8025	70.7256	*102.5750*
10	67.3966	68.0391	73.1985	76.0297	**78.4941**	78.1358	73.4817	69.7369	*102.9294*

Significant values are in bold and italics.

**Table 3:** CII values corresponding to the enhanced frames shown in Fig.2.

Item no	IFA ^27^	IVIF& CLAHE ^25^	ANV ^23^	NIFG ^26^	ZERO DCE ^38^	ZERO DCE++ ^39^	EFI NET ^40^	Flight NET ^41^	Proposed method
1	2.1383	2.0567	**2.3220**	2.1271	2.2670	2.2853	2.2857	2.2807	*2.8015*
2	2.1501	2.0651	**2.3377**	2.1352	2.2829	2.2970	2.2863	2.3231	*2.8110*
3	2.1857	2.0920	**2.3768**	2.2051	2.3316	2.3400	2.3081	2.4080	*2.8572*
4	2.2090	2.1092	**2.4090**	2.2275	2.3664	2.3704	2.3237	2.4146	*2.8835*
5	2.2624	2.1498	**2.5161**	2.2705	2.4383	2.4346	2.3609	2.4528	*2.9479*
6	2.2849	2.1686	**2.5500**	2.3381	2.4749	2.4674	2.3815	2.4090	*2.9696*
7	2.3374	2.3526	2.6123	**2.6636**	2.5422	2.5305	2.4201	2.4405	*3.0329*
8	2.3773	2.5272	**2.7685**	2.5640	2.5955	2.5825	2.4472	2.4722	*3.0843*
9	2.3637	2.3711	**2.7581**	2.5497	2.5891	2.5796	2.4548	2.4330	*3.0784*
10	2.3355	2.3482	2.4505	2.5066	**2.5554**	2.5483	2.4561	2.3819	*3.0396*

Significant values are in bold and italics.

where, $f_i$ is a feature vector of the distorted image, $\mu _r$ is a mean of the natural image feature distribution, $\sum _r$ is a covariance matrix of the natural image features, and $\sum _i$ is a covariance matrix of the distorted image features.

####  Blind/referenceless image spatial quality evaluator(BRISQUE)

Natural scene statistics (NSS) in the spatial domain are used by BRISQUE, a blind picture quality metric, to evaluate perceptual distortion. Unlike NIQE, it is trained on human-rated images, allowing it to detect common degradations such as noise, blur, and compression artifacts. It computes features from locally normalized luminance coefficients and applies a regression model—typically an SVR—to estimate quality. A lower score indicates better image quality. The metric can be expressed as^45^:

26 $$\begin{aligned} BRISQUE=R(z) \end{aligned}$$

where z is a feature vector derived from normalized luminance statistics, and R is a regression function trained on subjective quality scores.

**Table 4:** NIQE values corresponding to the enhanced frames shown in Fig.2.

Item no	IFA ^27^	IVIF& CLAHE ^25^	ANV ^23^	NIFG ^26^	ZERO DCE ^38^	ZERO DCE++ ^39^	EFI NET ^40^	Flight NET ^41^	Proposed method
1	7.0614	7.0020	6.7390	6.9925	6.1915	6.1760	3.8970	*3.0229*	**3.4165**
2	6.8226	6.7682	6.4216	6.8109	6.3899	6.2826	4.0527	*2.8483*	**3.6047**
3	6.7760	6.8372	6.5983	6.6384	6.1940	6.2931	4.2642	*2.7691*	**3.2585**
4	7.3590	7.3827	7.0487	7.3636	6.7057	6.6554	3.7856	*2.8450*	**3.3404**
5	6.6529	6.5575	6.5987	6.5619	6.2147	6.0993	4.0215	*2.8153*	**3.5194**
6	6.6678	6.5912	6.5601	6.7455	6.3951	6.2483	4.3027	*3.0269*	**3.5121**
7	6.8877	6.9255	6.7822	6.8816	6.2419	6.2695	4.3841	*2.9886*	**3.3775**
8	6.0748	6.0591	6.2821	6.1303	5.8318	5.8155	4.4271	*2.8820*	**3.1270**
9	6.4852	6.4914	6.5695	6.4220	5.9174	5.9425	4.0645	*2.7565*	**3.0570**
10	7.2695	7.2088	6.7978	7.0182	6.1561	6.0975	4.1823	*3.1442*	**3.3090**

Significant values are in bold and italics.

**Table 5:** BRISQUE values corresponding to the enhanced frames shown in Fig.2.

Item no	IFA ^27^	IVIF& CLAHE ^25^	ANV ^23^	NIFG ^26^	ZERO DCE ^38^	ZERO DCE++ ^39^	EFI NET ^40^	Flight NET ^41^	Proposed method
1	36.0067	36.1580	33.1396	35.8641	29.9194	29.7144	**20.7308**	27.0069	*5.9959*
2	36.5541	36.7819	34.3082	36.7005	30.4899	30.3224	**21.6255**	27.6644	*5.9864*
3	36.3290	36.5621	34.4002	36.6755	30.6826	30.6139	**20.6941**	27.5758	*9.5139*
4	36.7083	36.9601	34.4773	37.1666	31.4158	31.2043	**21.1058**	27.3235	*9.2099*
5	37.3570	37.5236	34.2759	37.3814	31.8145	31.6145	**21.2110**	27.5759	*14.5532*
6	37.0646	37.1505	34.0714	37.0813	31.6586	31.6110	**22.6467**	27.4718	*14.2379*
7	37.5922	38.1543	36.0347	37.9165	31.7955	31.8039	**20.2676**	26.1664	*14.5578*
8	37.6455	38.1650	34.9058	38.0620	32.1316	32.0091	**22.7614**	25.1380	*7.0469*
9	38.1254	38.4243	35.4031	38.4400	32.4989	32.6021	**22.0843**	24.8236	*6.1346*
10	37.9847	38.2495	35.9997	38.2984	32.8512	32.8133	**23.0151**	25.4058	*7.3625*

Significant values are in bold and italics.

**Fig. 3:**
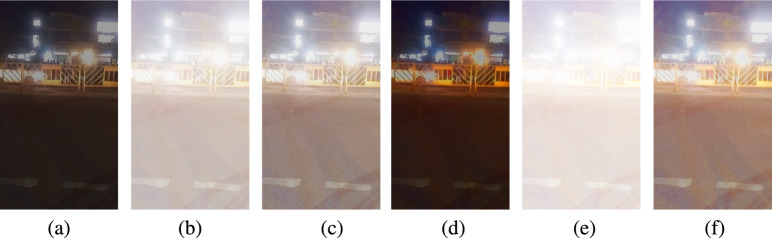
Ablation study results showing different variants of the proposed enhancement framework. Subfigures (**a**–**f**) correspond to Original, IVIFI without $\lambda - q$ tuning, IVIFI with $\lambda - q$ tuning, HVI transformation only, proposed method with IFI, and the proposed method, respectively.

### Ablation study

To assess the individual contribution of each component in our framework, we conducted an ablation study. The baseline case (without enhancement) results in low entropy and poor perceptual scores, highlighting the necessity of enhancement. Applying IVIFI with fixed parameters leads to a noticeable increase in entropy; however, the lack of adaptivity limits its overall effectiveness. By incorporating entropy-driven $\lambda$ – q tuning in the RGB space, the method achieves significant improvements in perceptual quality and structural consistency, as reflected by a reduction in NIQE and BRISQUE, along with an increase in entropy, AMBE, and CII. The HVI transform, when applied independently, enhances both contrast and color consistency; however, it falls short of delivering competitive performance on distortion-oriented metrics. When all modules are combined (IVIFI + $\lambda$ – *q* tuning + HVI), the proposed method consistently outperforms all intermediate variants and achieves the best performance across all objective metrics. These findings confirm that each module contributes positively to the overall enhancement, while their integration provides the most robust and balanced improvement. Representative visual results for frame 8 are shown in Fig.3, while the corresponding metric values for each component are reported in Table 6.

**Table 6:** Ablation study: quantitative evaluation of different variants of the proposed method Fig.3.

Metric	IVIFI (fixed $\lambda ,q$)	IVIFI ($\lambda ,q$) tuning	HVI	Proposed (IFI)	Proposed method
Entropy	5.7239	5.9963	6.6375	6.2085	7.0453
AMBE	154.7525	108.6631	0.5754	182.0601	102.7177
CII	4.1402	3.2049	1.0117	4.6943	3.0843
NIQE	4.9267	5.1472	5.7370	5.2684	3.1270
BRISQUE	26.0143	27.2031	16.0944	26.9696	7.0469

**Table 7:** Average metric comparison over all 60 frames corresponding to Fig. 2.

Metric	IFA ^27^	IVIF CLAHE ^25^	ANV ^23^	NIFG ^26^	ZERO DCE ^38^	ZERO DCE++ ^39^	EFI NET ^40^	Flight NET ^41^	Proposed
Entropy	7.1210	7.2278	6.6712	7.0722	6.7114	6.7043	7.2426	7.0622	7.1827
AMBE	81.5057	77.9790	**89.6019**	85.2503	78.6252	78.9536	70.2339	87.2849	*102.5844*
CII	2.9041	2.841	**3.0934**	2.9967	2.8212	2.8292	2.6202	3.0281	*3.3709*
NIQE	6.7597	6.7147	6.5810	6.6875	6.0014	6.0124	3.8952	*2.5507*	**3.0564**
BRISQUE	37.1886	37.4913	34.7439	37.4656	32.3713	32.2388	20.4186	**20.3622**	*10.9204*

Significant values are in bold and italics.

**Table 8:** Computation time (in seconds) for the enhanced frames shown in Fig. 2.

Item no	IFA ^27^	IVIF& CLAHE ^25^	ANV ^23^	NIFG ^26^	Proposed method
1	19.80	54.97	7.74	14.31	**4.16**
2	19.11	48.24	7.65	13.20	**3.79**
3	18.94	47.78	7.01	13.67	**3.67**
4	19.16	46.52	6.98	16.62	**3.80**
5	20.03	46.36	7.39	22.92	**5.52**
6	20.01	46.47	7.29	19.87	**4.00**
7	19.58	51.24	7.19	17.97	**4.09**
8	19.19	53.75	7.87	20.18	**3.97**
9	19.21	47.86	7.66	18.26	**3.90**
10	19.45	53.56	6.88	18.04	**3.83**

Significant values are in bold.

### Limitations

The proposed method using the interval-valued intuitionistic fuzzy generator (IVIFG) combined with HVI space is specifically designed to preserve and enhance the natural quality of low light video frames. It is not intended for increasing contrast or brightness alone.

## Conclusion and future work

In conclusion, this research proposes a novel and effective enhancement method for low-light video frames by integrating an interval-valued intuitionistic fuzzy generator (IVIFG) with the HVI color space. Unlike conventional methods that primarily focus on increasing contrast or brightness, this approach is specifically designed to preserve and enhance the natural appearance of low-light videos, especially in situations where reference frames are unavailable. An entropy-based selection strategy is employed to extract the most visually optimal frames, which are then compiled into the final enhanced video. The method’s performance is validated using no-reference quality metrics-entropy, AMBE, CII, NIQE, and BRISQUE - which clearly demonstrate its effectiveness over existing techniques. While the current method uses the spatial domain to preserve texture and visual consistency, future work will explore integrating deep learning and optimization techniques to further enhance quality and efficiency. Apply this approach to further complex situations, such as low-light videos affected by haze, underwater recordings, and medical endoscopic videos. The method has a lot of potential for practical uses like surveillance footage and imaging for health care.

## Data Availability

The low-light video data used in this study were generated by the authors using a Moto G84 smartphone camera and were created specifically for this research. The authors retain full copyright of the data. The videos are available from the corresponding author upon reasonable request.
